# Expression characteristics of piRNAs in ovine luteal phase and follicular phase ovaries

**DOI:** 10.3389/fvets.2022.921868

**Published:** 2022-09-08

**Authors:** Chunyan Li, Rensen Zhang, Zijun Zhang, Chunhuan Ren, Xiangyu Wang, Xiaoyun He, Joram Mwashigadi Mwacharo, Xiaosheng Zhang, Jinlong Zhang, Ran Di, Mingxing Chu

**Affiliations:** ^1^Key Laboratory of Animal Genetics, Breeding and Reproduction of Ministry of Agriculture and Rural Affairs, Institute of Animal Science, Chinese Academy of Agricultural Sciences, Beijing, China; ^2^College of Animal Science and Technology, Anhui Agricultural University, Hefei, China; ^3^Small Ruminant Genomics, International Center for Agricultural Research in the Dry Areas (ICARDA), Addis Ababa, Ethiopia; ^4^Institute of Animal and Veterinary Sciences, Animal and Veterinary Sciences, SRUC and Center for Tropical Livestock Genetics and Health (CTLGH), Midlothian, United Kingdom; ^5^Tianjin Institute of Animal Sciences, Tianjin, China

**Keywords:** sheep, piRNA, ovary, small RNA-seq, reproductive phase

## Abstract

PIWI-interacting RNAs (piRNAs), as a novel class of small non-coding RNAs that have been shown to be indispensable in germline integrity and stem cell development. However, the expressed characteristics and regulatory roles of piRNAs during different reproductive phases of animals remain unknown. In this study, we investigated the piRNAs expression profiles in ovaries of sheep during the luteal phase (LP) and follicular phase (FP) using the Solexa sequencing technique. A total of 85,219 and 1,27,156 piRNAs tags were identified in ovine ovaries across the two phases. Most expressed piRNAs start with uracil. piRNAs with a length of 24 nt or 27–29 nts accounted for the largest proportion. The obvious ping-pong signature appeared in the FP ovary. The piRNA clusters in the sheep ovary were unevenly distributed on the chromosomes, with high density on Chr 3 and 1. For genome distribution, piRNAs in sheep ovary were mainly derived from intron, CDS, and repeat sequence regions. Compared to the LP ovary, a greater number of expressed piRNA clusters were detected in the FP ovary. Simultaneously, we identified 271 differentially expressed (DE) piRNAs between LP and FP ovaries, with 96 piRNAs upregulated and 175 piRNAs downregulated, respectively. Functional enrichment analysis (GO and KEGG) indicated that their target genes were enriched in reproduction-related pathways including oocyte meiosis, PI3K-Akt, Wnt, and TGF-β signaling pathways. Together, our results highlighted the sequence and expression characteristics of the piRNAs in the sheep ovary, which will help us understand the roles of piRNAs in the ovine estrus cycle.

## Introduction

PIWI-interacting RNAs (piRNAs), are a class of newly discovered small non-coding RNAs with approximately a range from 24 to 32 nucleotide (nt) in length, which associates with PIWI proteins to constitute the core of piRNA pathways across animal species (1–4). PIWI proteins belong to a clade of the Argonaute protein family, which are mainly expressed in germ lines (5, 6). The piRNA-PIWI complexes are involved in multiple physiological regulations in germ cells, such as proliferation, meiosis, and anti-apoptosis (7, 8). It has been reported that piRNAs might protect genomic integrity from threat by “genomic parasites” -transposable elements (TEs) (9), and mediate the degradation of a large and diverse set of mRNAs and lncRNAs in spermatogenic cells (10). In model animals like *C. elegans* and zebrafish, piRNAs play important role in both spermatogenesis and oogenesis (7, 11). In other mammals such as macaques and humans, piRNA-like small RNAs are also enriched in the gonads and have similar characteristics (7).

The female estrus cycle includes the luteal phase (LP) and follicular phase (FP). Previous studies suggested that miRNAs play an important regulatory role in the reproductive phase transition in the ovaries of sheep and goats (12, 13). As another small non-coding RNA, a large number of piRNAs and several important interacting PIWI proteins were detected in sheep ovaries using RNA-seq and immunohistochemistry, respectively (14, 15). Combined with its function in regulating gene expression, piRNAs might exhibit expression characteristics and roles during different reproductive phases in the ewe estrus cycle.

Small-Tailed Han (STH) sheep, a famous indigenous breed in northern China, has attracted much attention for its excellent traits such as year-round estrus, precocious puberty, and high fecundity (16, 17). Therefore, it has been used as a typical breed for sheep reproduction studies (18–20). In the present study, small RNA expression profiles were obtained using RNA-Seq technology, then potential piRNAs were screened, and the expression characteristics of piRNAs in ovaries of STH sheep during LP and FP would be summarized. Additionally, differentially expressed piRNAs between LP and FP ovaries were screened and the function of their target genes would be predicted. Together, these results will be helpful for better understanding the sequence characteristics of piRNAs in the ovine ovary and the regulative role in the ovine estrus cycle.

## Methods

### Ethics statement

All the animals were authorized by the Science Research Department (in charge of animal welfare issues) of the Institute of Animal Science, Chinese Academy of Agricultural Sciences (IAS-CAAS). The sample collection was approved by the animal ethics committee of IAS-CAAS (No. IAS2019-49).

### Animals pretreatment and sample collection

Twelve STH ewes, which were consistent in age (3 years old) and weight (55–60 kg) from the same farm, were selected and raised under the same condition at Yuncheng Chenglian Sheep Industry Co., Ltd. (Yuncheng, China). They were processed by estrus synchronization with a Controlled Internal Drug Releasing device (CIDR, progesterone 300 mg, Inter Ag Co., Ltd., New Zealand) for 12 days. Within 45–48 h (follicular phase, FP) after CIDR removal, 6 ewes were slaughtered. The remaining 6 ewes were slaughtered on day 7 (luteal phase, LP) after CIDR removal (21). After slaughtering, ovarian samples were immediately collected and stored in liquid nitrogen.

### RNA extraction of sheep ovaries samples

The total RNA of twelve samples was extracted using TRIzol reagents (Invitrogen, Carlsbad, CA, USA) according to the manufacturer's instruction. A total of 1% agarose gels were used to monitor whether isolated RNA was degraded or contaminated. Quality, concentration, and integrity of RNA were assessed by NanoPhotometer^®^ spectrophotometer (Implen, CA, USA), Nano 6000 assay kit of the Agilent Bioanalyzer 2100 system (Agilent Technologies, CA, USA), and Qubit^®^ RNA assay kit in Qubit^®^ 2.0 Fluorometer (Life Technologies, CA, USA), respectively. Among them, we only received total RNA samples with OD260/280 >1.7, OD260/230 <2.0, concentration ≥ 300 ng/μl, and integrity ≥ 7 for further analysis (22).

### Construction and sequencing of small RNA library

Each of twelve RNA samples (FP, n = 6; LP, n = 6; 3 μg of each) was used as input material for small RNA libraries. A FlashPAGE fractionator (Ambion, Life Technologies, Paisley, UK) was used to isolate small RNAs of <40 nt from total RNA. Sequencing libraries were generated using NEBNext^®^ Multiplex Small RNA Library Prep Set for Illumina^®^ (NEB, Ipswich, MA, USA) according to the manufacturer's instruction, index codes were added to attribute sequences to each sample. In brief, NEB 3′ SR Adaptor was directly, and specifically ligated to the 3′ end of miRNA, siRNA, and piRNA. After a 3′ ligation reaction, the SR RT Primer hybridized to the excess of 3′ SR Adaptor (that remained free after the 3′ ligation reaction) and transformed the single-stranded DNA adaptor into a double-stranded DNA molecule. This step is important to prevent adaptor-dimer formation. Besides, dsDNAs are not substrates for ligation mediated by T4 RNA Ligase 1 and, therefore, do not ligate to the 5′ SR Adaptor in the subsequent ligation step. 5′ ends adapter was ligated to 5′ ends of miRNAs, siRNA, and piRNA. Then first strand cDNA synthesis using M-MuLVA Reverse Transcriptase (RNase H^−^). PCR amplification was performed using LongAmp Taq 2X Master Mix, SR Primer for Illumina, and index (X) primer. PCR products were purified on 8% polyacrylamide gel (100 V, 80 min). The 140–160 bp products (the length of small non-coding RNA plus 3′ and 5′ adaptors) were recovered and dissolved in an 8 μl elution buffer. Finally, cDNA library quality was assessed on the Agilent Bioanalyzer 2100 system (Agilent Technologies, CA, USA) with DNA High Sensitivity DNA Chips (Agilent Technologies, Palo Alto, CA, USA). Single-end sequencing of 50 bp was then performed on an Illumina Hiseq 2500 platform (23).

### Small RNA annotation and piRNA identification

The obtained raw data of small RNA were filtered to remove the following reads: low-quality reads (reads with a base number of Qphred ≤ 5 accounting for more than 50% of the entire read length), reads with N content >10% (N Refers to the specific information of a base in a read sequence that cannot be determined at a certain position), reads containing 5′ adapter contamination, reads without 3′ adapter sequence and insert, reads containing poly A/T/C/G (The poly A/T/C/G may be due to sequencing errors). Then the 3′ adapter sequence and those <24 nt and >33 nt reads were removed from the remaining data (1–4, 24–26), and clean reads were obtained. The clean reads were aligned to the reference genome *Ovis aries v4.0* (27) using Bowtie (version 0.12.9). Perfectly mapped reads were filtered by following steps to remove tags: mapped to the miRbase v21.0 (http://www.mirbase.org/) to exclude conserved miRNAs, screened against RepeatMasker (http://www.repeatmasker.org/) and Rfam (http://rfam.sanger.ac.uk/search/) with Bowtie to filter sequences originating from protein-coding genes, rRNA, tRNA, snRNA, snoRNA, and repeats. The new miRNAs in the rest of the reads were predicted by Mireap 0.2, and then they were also removed. Finally, the remaining reads with 1U or 10A were filtered out as candidate piRNA.

### Prediction of piRNA cluster*s*

PiRNA clusters (pi-clusters) are particular genomic sites with piRNA-containing regions. We predicted potential pi-clusters harboring a minimum of 10 different piRNAs on sense and anti-sense strands of a chromosome with a scanning window of 5 kilobase length (In the same piRNA cluster, the distance between two windows cannot exceed 20 K).

### Differential expression of piRNAs between LP and FP ovaries

The expression levels of piRNAs were calculated and shown with a transcript per million (TPM) value. The differentially expressed (DE) piRNAs between two groups (LP vs. FP) were screened using DESeq R package (1.12.0) with *P-*value < 0.05, expression multiple > 2.0, and read counts > 10, simultaneously.

### Prediction and enrichment analysis of target genes of DE piRNA

The target genes of DE piRNAs were predicted by miRanda (v3.3a), RNAhybrid, and TargerScan, simultaneously (28, 29). Then, the GOseq R Bioconductor package was used to perform GO (http://www.geneontology.org/) enrichment analysis of the target genes (30). KOBAS_2.0 software (http://kobas.cbi.pku.edu.cn/index.php, USA) was used to test the statistical enrichment of the target gene candidates in KEGG (http://www.genome.jp/kegg/) pathways (31).

### Q-PCR detection of several candidate piRNAs and genes

Using qPCR detection technology, the expression levels of two candidate piRNAs (t00034730 and t00050151) and three target genes (SMAD2, ESR1, and ITPR2) in the follicular and luteal phases were analyzed. The sequences of reverse transcription stem-loop primers and qPCR amplification primers were shown in Supplementary Table S1. U6 was used as an internal reference gene. Complimentary DNA (cDNA) was synthesized from 1 μg of total RNA using the PrimeScript RT reagent kit (TaKaRa, Dalian, China). Q-PCR was performed, using a standardized protocol, with the ABI7500 system (Applied Biosystems, CA, USA). The 20 μl amplification reaction mixture was composed of 2.0 μl cDNA (at a 1:4 dilution) as a template, 10 μl of SYBR Premix Ex TaqTM II (TaKaRa), 1.6 μl of a gene- or miR-specific primer set (0.8 μl of each forward and reverse primer), 0.4 μl of ROX Dye II, and 6 μl of water. The reactions were incubated at 95°C for the 30 s, followed by 40 cycles of 95°C for 5 s and 60°C for 34 s, 12°C forever. The abundance of selected miRNAs was normalized relative to that of the U6 snRNA. All reactions were performed in triplicate. The threshold cycle (CT) was determined using the default threshold settings, and the data were analyzed with the 2^−Δ*ΔCt*^ method.

## Results

### Number of expressed piRNA in sheep ovaries during follicular and luteal phases

Deep sequencing, the result showed that 145,106,213 clean reads of small RNAs were expressed in the ovaries of STH sheep during the follicular phase (FP) and luteal phase (LP) (Table 1). After the following types of RNA including mRNA, rRNA, tRNA, snRNA, snoRNA, miRNA, and newly predicted miRNA sequences were filtered out from the reads that aligned to the genome, a total of 876,102 candidate piRNA reads were predicted. Specifically, 478,619 and 397,483 piRNA reads existed in sheep ovaries during FP and LP, respectively. After fusing the same reads into a single tag, a total of 127,156 and 85,219 tags (or unique reads) were acquired in FP and LP ovaries, respectively.

**Table 1:** Summary of sequencing data of small RNA and piRNAs in ovine ovaries.

**Phases**	**Total reads**	**Clean reads**	**Small RNA**	**Mapped**	**miRNA**	**tRNA**	**rRNA**	**piRNA**	**piRNA**
	**(<40 nt)**	**(<40 nt)**	**reads (18–35 nt)**	**small RNA**	**reads**	**reads**	**reads**	**reads**	**tags**
FP	76,529,285	75,205,273	71,756,571	66,454,144	48,649,422	952,102	896,005	478,619	127,156
LP	71,805,605	69,900,940	67,217,098	59,049,777	40,482,038	1,943,295	742,563	397,483	85,219

FP, follicular phase; LP, luteal phase.

### Characteristics of piRNA sequences in sheep ovary

**Figure 1:**
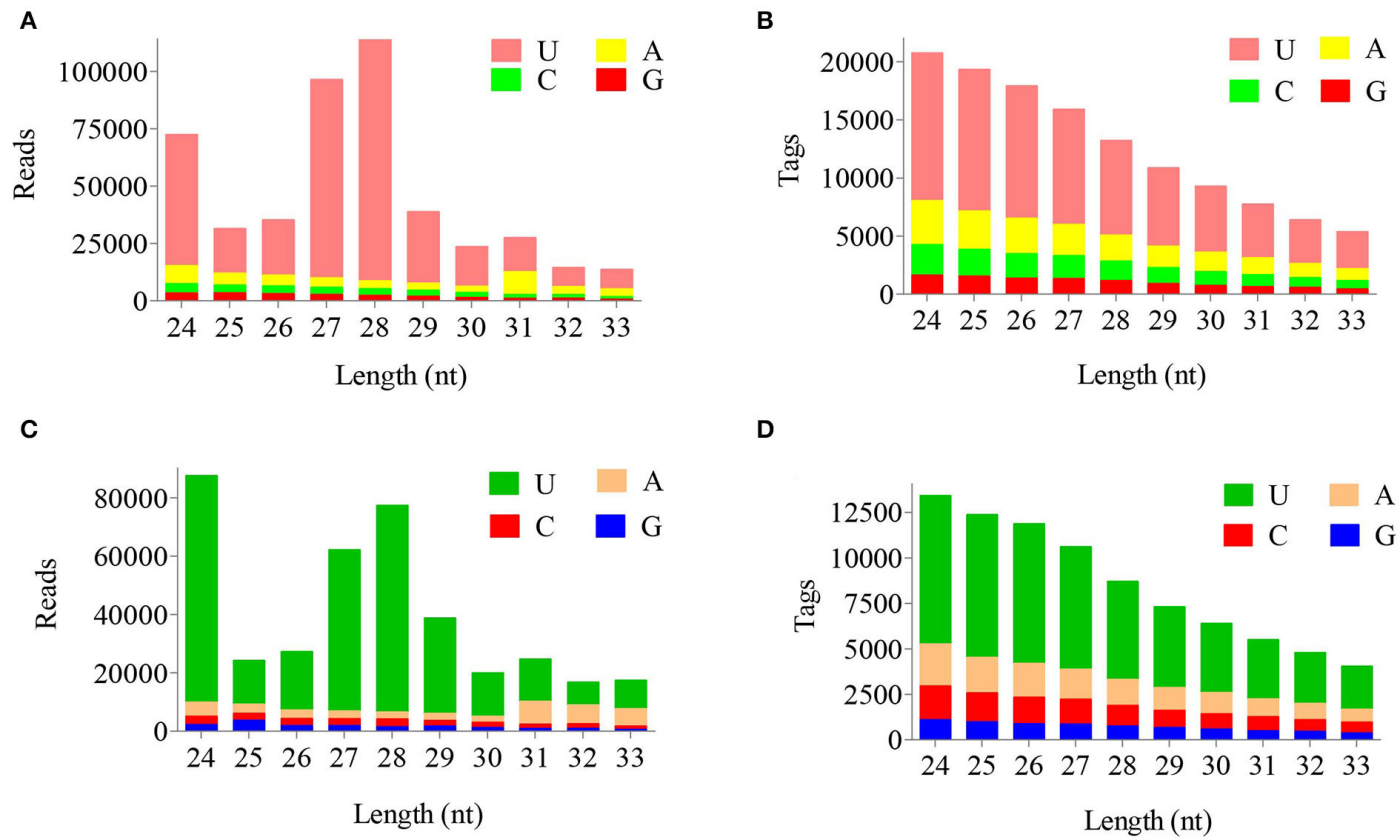
Length-wise distribution of piRNAs in two groups. Length distribution of piRNA reads **(A)** and the tags **(B)** in FP. Length distribution of piRNA reads **(C)** and the tags **(D)** in LP.

**Figure 2:**
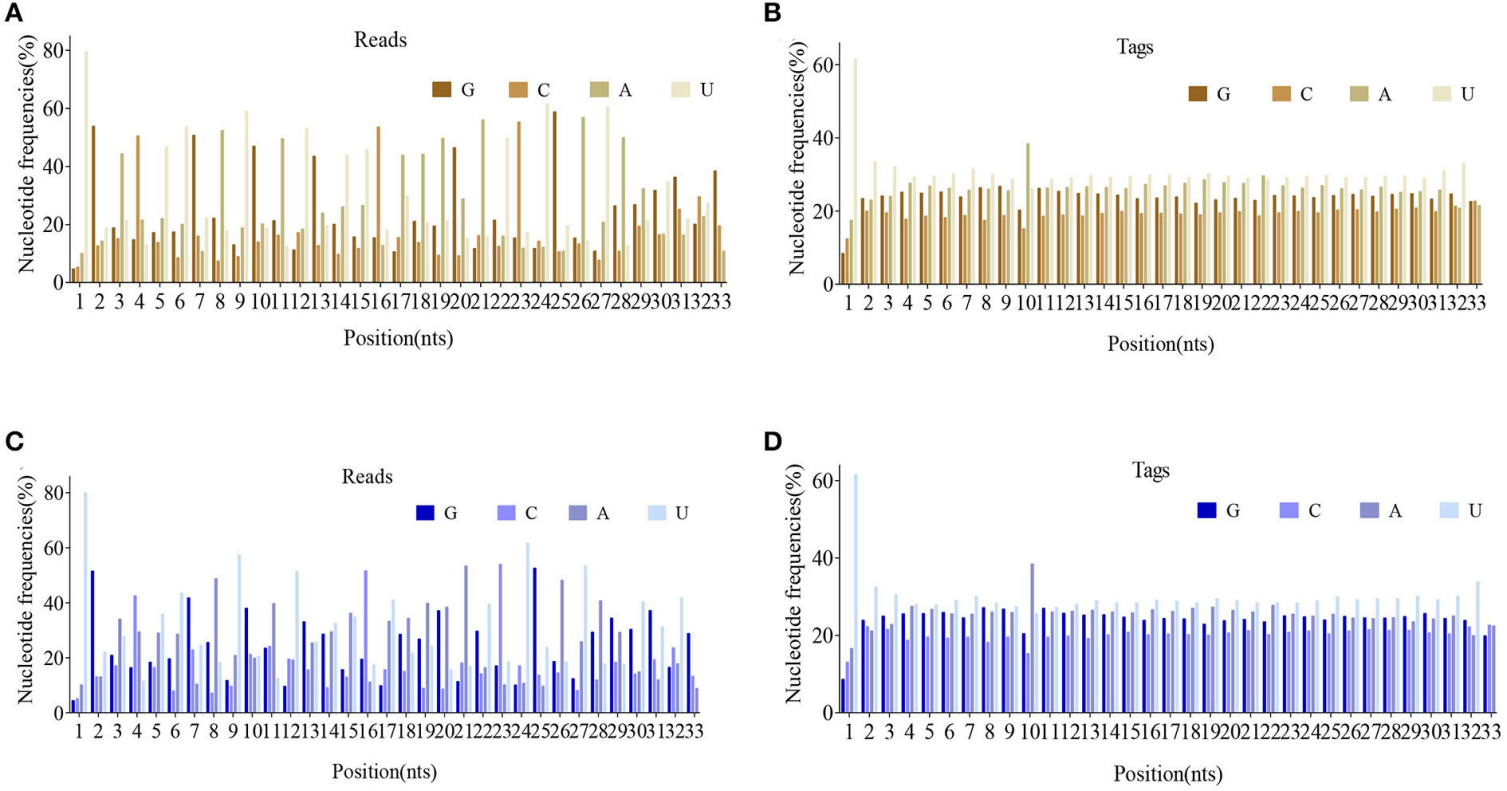
5′-3′ positional nucleotide frequencies of piRNAs in two groups. piRNA base bias **(A)** and the unique base bias **(B)** in FP. piRNA base bias **(C)** and the unique base bias **(D)** in LP.

The reads and tags length of ovarian candidate piRNAs during FP and LP were statistically summarized in Figure 1 (data in Supplementary Table S2). The results indicated that length distributions of the reads and tags were similar in both reproductive phases. Peak values of reads number appeared at 24 nt and 27–29 nts (Figures 1A,C) during both phases, and the number of tags showed a decreasing distribution between 24 and 33 nts (Figures 1B,D). More than 75% of candidate piRNAs start with uracil. Besides, the base distribution frequency of each position in piRNA reads and tags was also statistically summarized during FP and LP and was shown in Figure 2 (data in Supplementary Table S3).

### Chromosomal and genomic origins of piRNAs in ovary of sheep

**Figure 3:**
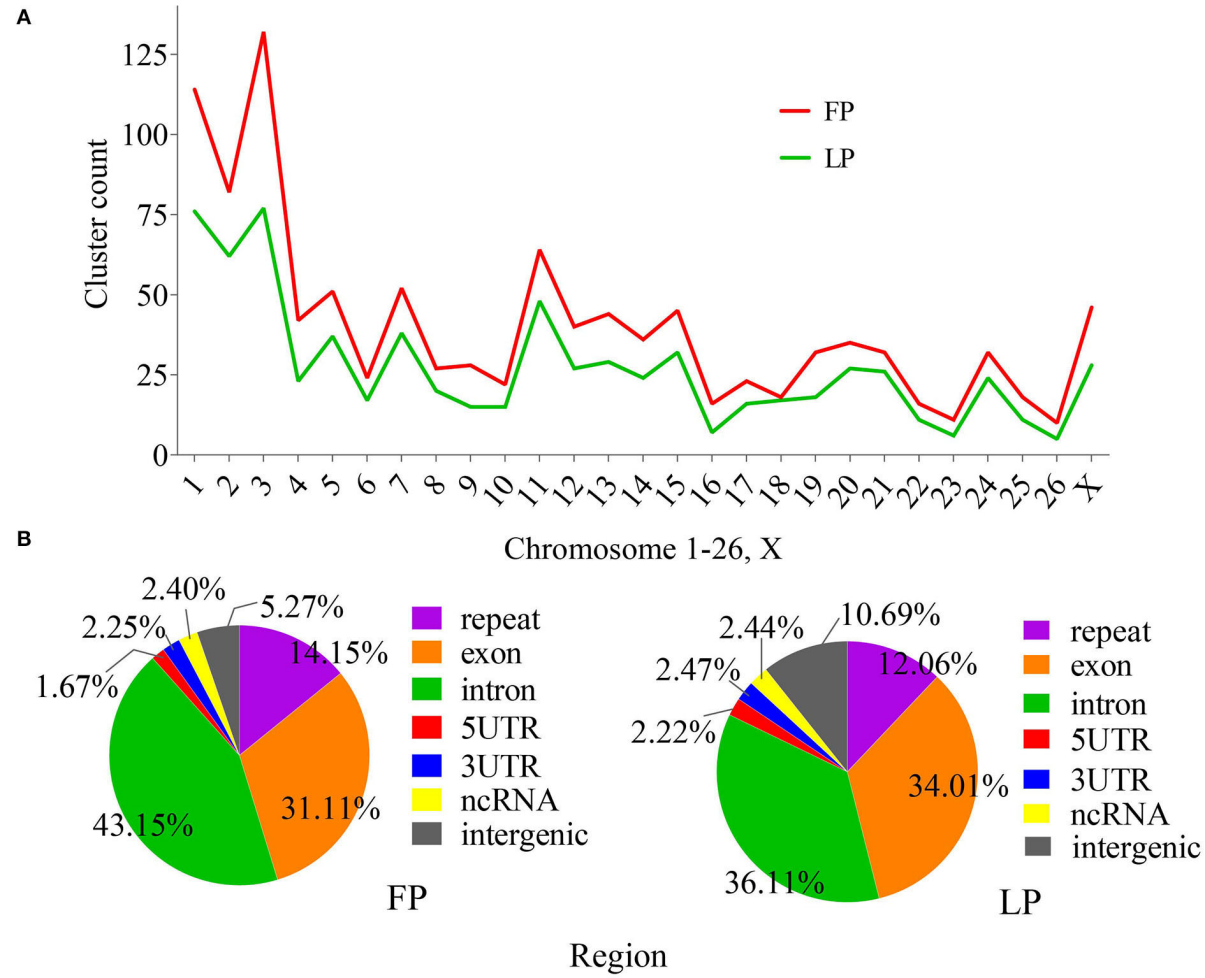
Pi-clusters across various chromosomes **(A)** and genomic regions of matched piRNAs falling within the pi-clusters **(B)** in FP and LP groups.

PiRNAs are processed in a Dicer-independent manner, mostly from the genomic piRNA clusters (pi-clusters). These clusters produce most piRNAs which control the transposable element (TE) activity. Searching on a scanning window of length 20 kilobase (kb) with a window shift of 5 kb, 1,092, and 736 highly dense pi-clusters were identified in FP and LP ovaries, respectively (Supplementary Table S4). The pi-clusters numbers in the FP ovary were greater than that in the LP ovary. We found that the number of piRNAs on chromosomes in ovine ovaries was not proportional to chromosome length but correlated with the number of genes. The high cluster density was observed on chromosomes 3 and 1, whereas only a few clusters existed on chromosomes 16, 23, and 26. Meanwhile, 46 and 28 pi-clusters were detected on chromosome X in FP and LP ovaries, respectively (Figure 3A).

In addition, the genomic source of piRNA was also analyzed. The distribution proportion of piRNAs in the intergenic region was higher during LP than that during FP, and the distribution proportion of piRNAs in other genomic regions was not significantly different between FP and LP (Figure 3B). These piRNAs were mainly aligned to the intron, CDS, and repeat sequence regions. However, the piRNAs that aligned the 5'UTR, 3'UTR, and lncRNA regions were relatively less.

**Figure 4:**
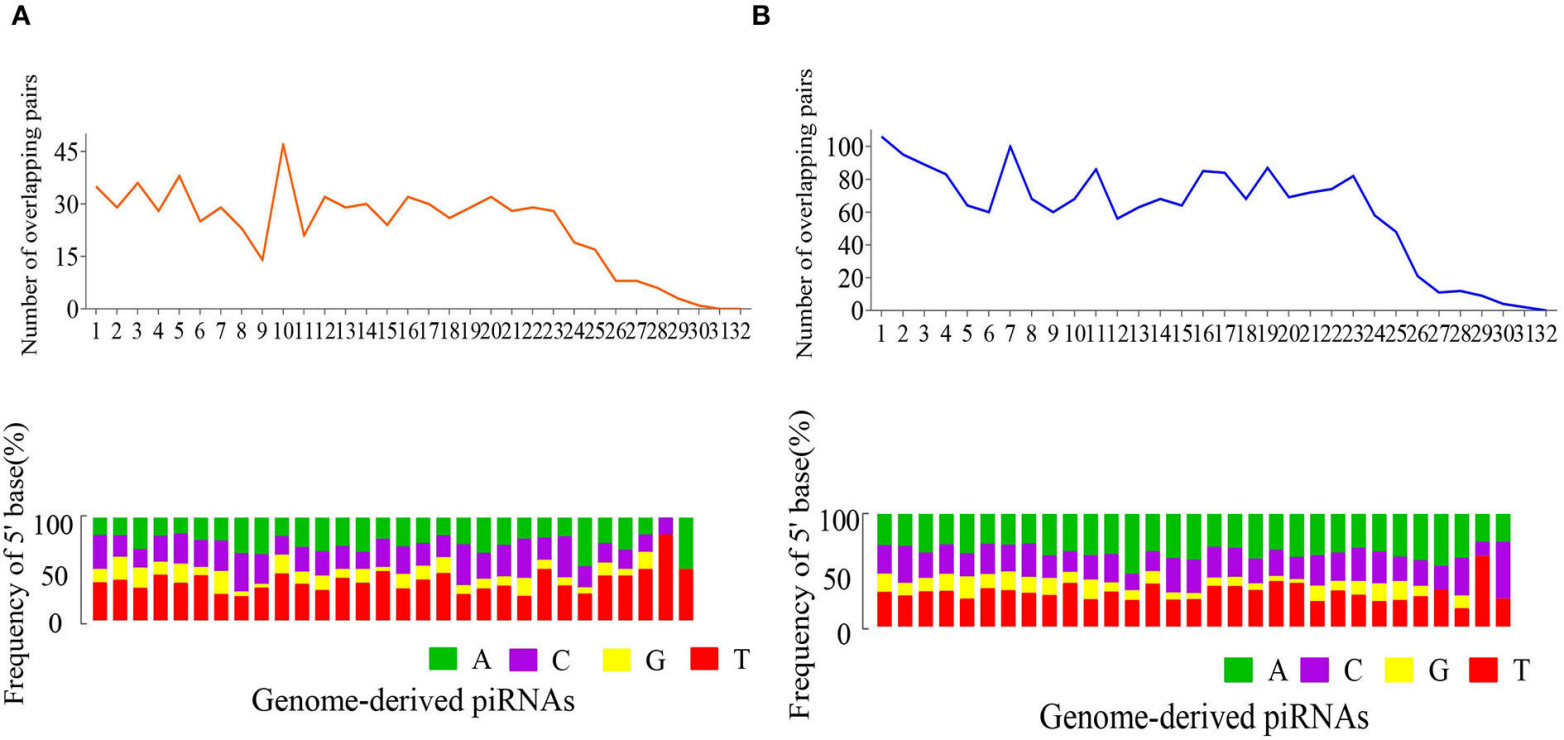
Overlapping pairs and the 5′ base frequencies of genome-derived piRNAs in FP **(A)** and LP **(B)**.

The ping-pong signature for genome derived piRNAs was detected (Figure 4, Supplementary Table S5). The reads exhibited an obvious ping-pong signature in the FP ovary (Figure 4A), i.e., there was a peak in the number of the overlapping pairs at the position of the 10^th^ base from the 5' end (Figure 4B).

**Figure 5:**
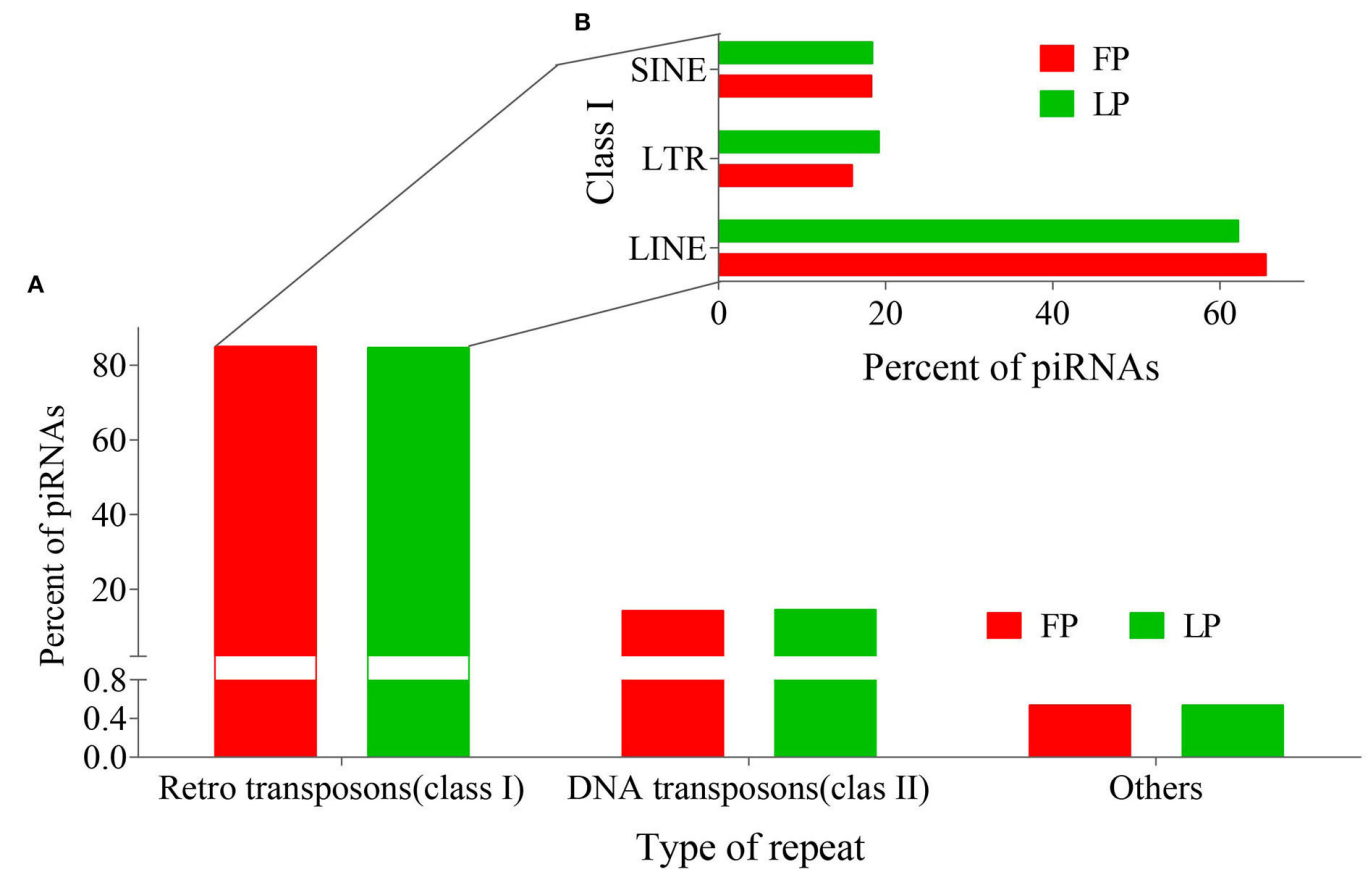
Distribution of origin of FP and LP-piRNAs among **(A)** Repeats and **(B)** Retro repeats.

Transposable elements (TEs) are usually recognized as a cause of intra-genomic variation as well as diversification, also serving as the origin of piRNAs. Reciprocally, piRNAs regulate transposon silencing. Therefore, we searched for piRNAs origin in all classes of TEs, including Retrotransposons (Class I: SINE, LTR, LINE), DNA transposons (Class II), and others (Supplementary Table S6). Results showed that most piRNAs originated from class I TEs in both FP and LP ovaries (Figure 5A). Among these, LINE repeats harbored the highest number of piRNAs (Figure 5B).

### Analysis of DE piRNA and their target genes between FP and LP ovaries of sheep

**Figure 6:**
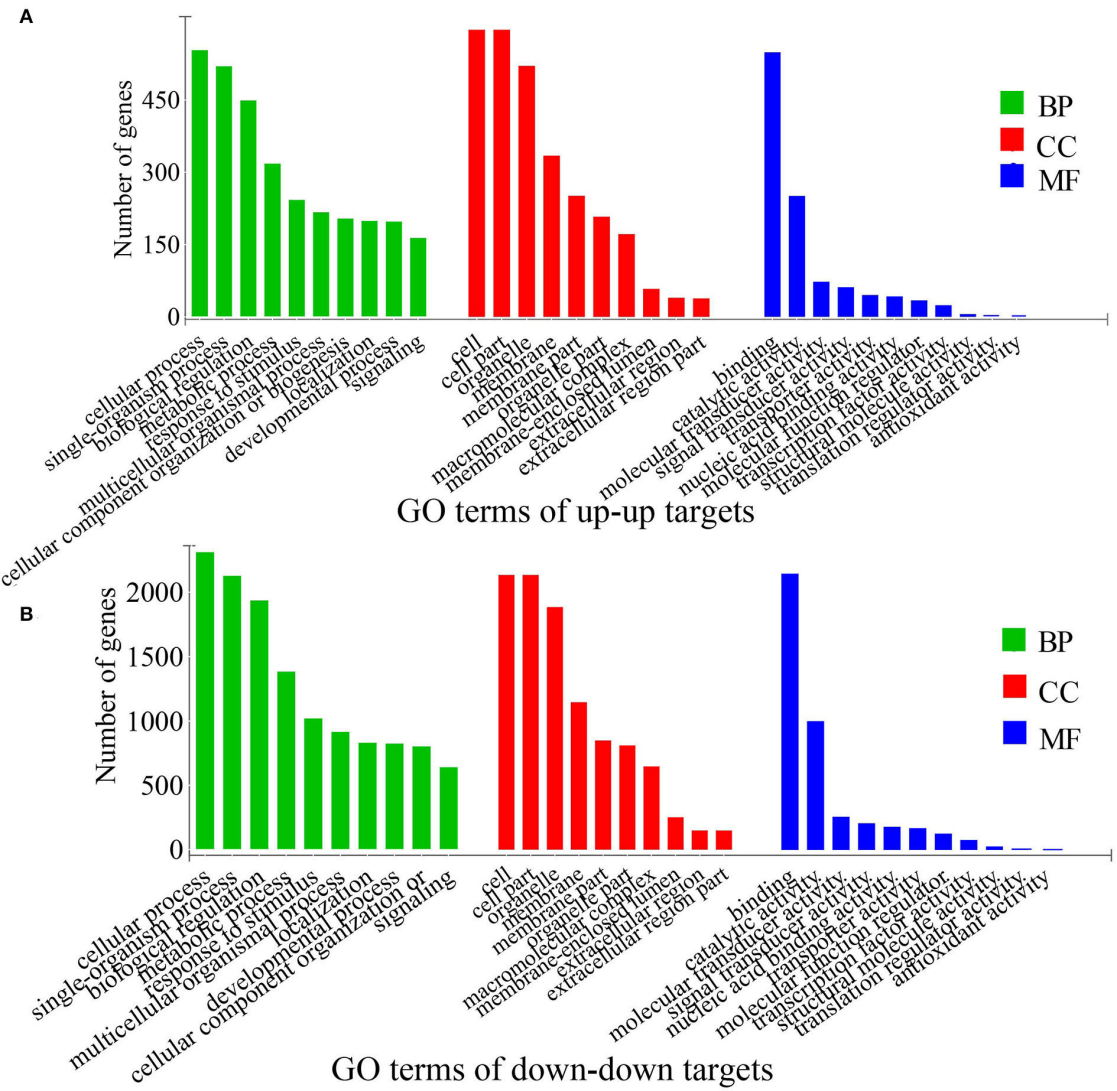
Top 10 GO enrichment of the differentially expressed target mRNAs in LP vs. FP. **(A)** Top 10 GO terms of up-up regulated targets, **(B)** top 10 GO terms of down-down regulated targets.

**Figure 7:**
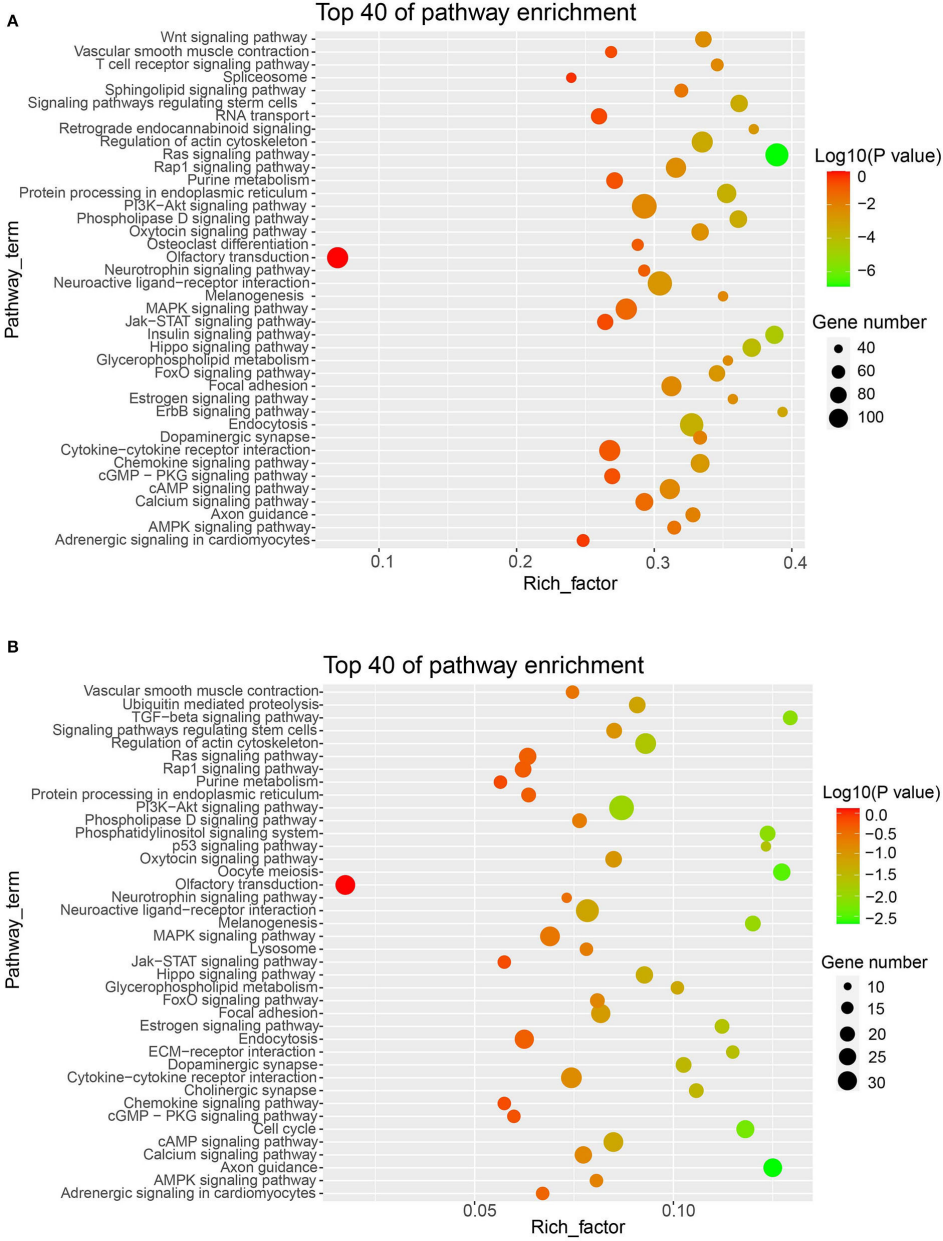
Top 40 of KEGG enrichment of the up-up regulated target mRNAs **(A)** and the down-down regulated target mRNAs **(B)** in LP vs. FP.

Between two reproductive phases (FP and LP), a total of 271 differentially expressed (DE) piRNAs were detected in the ovine ovary, with 175 upregulated piRNAs in FP and 96 upregulated piRNAs in LP, respectively (Supplementary Table S7). Then, we predicted the target mRNAs of these DE piRNAs (Supplementary Tables S8, S9). To understand the biological process and physiological function associated with DE piRNAs in the estrous cycle, functional enrichment analyses were performed for the target mRNAs of DE piRNAs. GO annotation revealed that the targets were specifically enriched in biological rhythms, reproduction, signal transducer, and antioxidant terms (Figure 6, Supplementary Table S10). KEGG pathway results indicated that the target genes of upregulated piRNAs were mainly enriched in PI3K-Akt signaling, Ras signaling, cAMP signaling, insulin signaling, and Wnt signaling pathways (Figure 7A, Supplementary Table S11). The targets of downregulated piRNAs were enriched in TGF-β signaling, PI3K-Akt signaling, oocyte meiosis, p53 signaling, hippo signaling, calcium signaling, cell cycle, and cAMP signaling pathways (Figure 7B, Supplementary Table S11). The relative expression level of several candidate piRNAs and predicted target genes were shown (Supplementary Figure S1, Supplementary Table S12) using qPCR technology. The expression of t00034730 was negatively correlated with those of SMAD2 and ESR1, and the expression of t00050151 was negatively correlated with that of ITPR2, which suggested target relationships between them.

## Discussion

PiRNAs, as epigenetic regulatory factors, can combine PIWI proteins to form a conserved piRNA-induced silencing complex (piRISC). PiRISC may recognize target nascent TE mRNAs, initiate transcriptional gene silencing, and ensure genome integrity (32, 33). piRNAs have been initially detected in *Drosophila* germline cells (34), and they are generated by two coupled pathways: the primary pathway and the secondary pathway. The primary pathway involves transcription of pi-clusters, nuclear processing of pi-precursors, and piRNA maturation. The secondary pathway consists of the ping-pong cycle, which in turn triggers the generation of primary piRNAs (29). In ovarian germ cells, piRNAs mainly originate from dual-strand clusters, they can combine with three kinds of PIWI proteins (Piwi, Aubergine, and Ago3) to silence transposons and guarantee oogenesis (35). They comprise 24–32 nts sequences, with phosphorylated 5′ ends and 2′-O-methylated 3′ ends.

In mammals, piRNAs are predominantly detected in testicular development and spermatogenesis (22, 36, 37). Most postnatal piRNAs in developing testis are produced from a limited number of piRNA clusters and divided into two types depending on the stage of their expression: prepachytene clusters arising from gene sequences, and pachytene clusters from the intergenic origin that are likely functionally redundant (38–40). A previous study of piRNAs indicated that potential piRNAs (18–30 nts) have a bimodal length distribution, with two peaks at 22 nt and > 28 nt in sexually immature and mature yak testes, and differential target genes are involved in spermatogenesis through ECM-receptor interaction and PI3K-Akt signaling pathways (41). Horse testicular putative piRNAs are in the length of 26–32 nts, with a strong preference for uridine (U) at the 5′ end and for adenine (A) at the 10^th^ position. The candidate piRNAs in porcine testes with length are predominantly 28 nt, and their target genes involve testicular development through the ECM-receptor interaction, focal adhesion, Wnt, and PI3K-Akt signaling pathways (42). Interestingly, piRNA reads from immature testis are located in the intergenic and intron regions, the reads from mature testis are widely distributed in the CDS region, and a few are in intron and intergenic regions (22, 42). We found that piRNAs in the ovary of sheep are mainly aligned to the intron and CDS regions. Besides, piRNAs in the ovaries of Chinese hamsters (43), mice, and humans (44) act as regulating transposon activity. The putative piRNA-generating genes enrich progesterone-mediated oocyte maturation, glycerolipid metabolism, oocyte meiosis, and glycerophospholipid metabolism pathways (22). These results possibly hint that ovarian piRNAs mainly play roles in inhibiting abnormal transposon expression and regulating gene expression, to ensure the integrity and correctness of the oocyte genome and normal physiological activities for oogenesis.

The piRNA in both antisense and sense orientations and the 5' ends of piRNAs display a uridine preference, which is generated *via* a self-reinforcing amplification cycle, also called the ping-pong mechanism (4, 29, 45). Recent studies of piRNAs have been subjected to extensive research in the Drosophila ovary for their roles in repressing TEs in germ cells (fGS) and somatic cells (OSS and OSC). These ovary-derived cell lines exhibit ping-pong piRNA signatures, with high expression of several germline pi-clusters in fGS and OSS cells, the piRNA loci in genomic segments with a dominant and relatively high density of 24–30 nts reads, most of these loci generated piRNAs with >60% uridine bias. Ovarian germ cells have both primary piRNAs and secondary piRNAs generated by the cleavage of complementary target transcripts, usually in the service of TEs defense (46). However, the pi-clusters are more active in germ stem cells and their early progeny than late germ cells and exhibit more expressed numbers of ping-pong signatures. The higher piRNA levels in germ stem cells and early progeny can be attributed to the above pi-clusters, and UTRs of the target mRNAs may produce sense, antisense, or dual-stranded piRNAs (47). Notably, our data suggest that ovarian piRNAs originate from pi-clusters enriched in intron and CDS regions, with a length of 24–33 nts and peaks at 24, 27–29 nts. Most piRNAs start with uracil, showed a ping-pong signature during the follicular phase in sheep, for overlapping pairs showed 10 nt 5′ overlaps of putative piRNAs with opposite orientation. Previous studies have shown that the sequences of piRNAs in testis tissue have distinct ping-pong characteristics, implying that piRNAs in testis play an important role in inhibiting transposon activity (48). However, the ping-pong signal of piRNAs in the sheep ovary, especially in the luteal phase, is weak, which suggests that piRNAs in the ovary mainly play other biological roles in addition to partially inhibiting transposon activity, such as regulating gene expression, which is consistent with its distribution ratio in coding genes is much higher than that in testis.

Previous studies suggested that miRNAs play an important regulatory role in the reproductive phase transition in the ovaries of sheep and goats (12, 13). As another small non-coding RNA, a total of 166,164 candidate piRNAs that originate from 1,251 clusters were predicted in sheep ovaries by de novo prediction and homology search after Solexa-Seq (14). Additionally, several kinds of important piRNAs-interacting PIWI proteins (PIWIL1, PIWIL2, PIWIL4, and AGO3) were detected in sheep germ cells using the immunohistochemistry method, especially in oocytes of mature follicles (15). Compared with its function in regulating gene expression, these results implied that piRNAs might exhibit distinct expression characteristics and roles during each reproductive phase in the estrus cycle. Indeed, we found different expression patterns and a great deal of DE piRNAs between follicular and luteal phases.

Functional assessment analysis implied that the targets of DE piRNAs between FP and LP ovaries enriched in reproduction related pathways, such as oocyte meiosis, cAMP, PI3K-Akt, hippo, p53, Wnt, and TGF-β signaling pathways. Usually, in mammalian oocytes, the meiosis starts during embryogenesis and concludes at fertilization (49). Meiotic prophase arrest is maintained a long time before follicles reached the preovulatory stage, then meiotic resumption to achieve follicles grow to the preovulatory stage, which is affected by multiple signals. During follicle development, FSH upregulates TGF-β signaling molecules in mural granulosa cells (MGCs). TGF-β increases the levels of natriuretic peptide type C (NPPC) secreted by MGCs and maintains oocyte meiotic arrest *via* the activation of guanylyl cyclase-linked natriuretic peptide receptor 2 (NPR2) (50). Besides, cAMP maintains meiotic arrest by suppressing the activation of maturation promoting factor (MPF) in the luteal phase. Whereas LH signaling can decrease the level of cAMP, and induces MPF activation, resulting in germinal vesicle breakdown (GVB) and meiotic resumption in the follicular phase (49). Wnt signaling is an evolutionarily conserved pathway that negatively regulates follicular development through modulating the expression of transcription factor Forkhead box O3a (Foxo3a) signaling components (51). It is currently established that Foxo3a can enhance transcriptional regulation of its target genes, thereby inducing oocyte apoptosis, and inhibiting the activation of primordial follicles. Namely, Wnt signaling reduces the number of primordial follicles transformed into mature follicles, thus preserving the follicular reserves (52). In terms of individual genes, we found that several piRNAs tend to be negatively correlated with genes regulating granulosa cell proliferation and follicle development. During the follicular phase, the increased expression of SMAD2 can promote the proliferation of granulosa cells and the further development of follicles. In addition, after receiving estrogen stimulation signals, genes such as ESR1 and ITPR2 are required to participate in the activation of downstream genes and the proliferation of granulosa cells. piRNAs are predicted to repress the expression of the above three genes (SMAD2, ESR1, and ITPR2) during the luteal phase and abolish repression for them during the follicular phase. Combined with the above studies, our results suggest that the roles of these DE piRNAs are mainly involved in the regulation of follicular growth, development, and ovulation.

## Conclusion

A total of 127,156 and 85,219 piRNAs tags were identified in ovine ovaries during FP and LP, respectively. The piRNAs exhibited an obvious ping-pong signature in the ovary of FP. The piRNAs clusters were unevenly distributed on the chromosomes, with high density on chromosomes 3 and 1. piRNAs in the ovine ovary were mainly derived from intron, CDS, and repeat sequence regions. Among the transposable components, LINE repeats harbored the highest number of piRNAs. Additionally, we identified 271 DE piRNAs between LP vs. FP ovaries, including 96 upregulated and 175 downregulated piRNAs. Their target genes were enriched in reproduction-related pathways including oocyte meiosis, PI3K-Akt, Wnt, cAMP, and TGFβ signaling pathways. Collectively, our findings provided useful information for further revealing the roles of ovarian piRNAs in the ovine estrus cycle.

## Data availability statement

Raw data of sequencing have been submitted to the repository, the bioproject accession number to cite for these SRA data in NCBI is PRJNA831363.

## Ethics statement

All the animals were authorized by the Science Research Department (in charge of animal welfare issues) of the Institute of Animal Science, Chinese Academy of Agricultural Sciences (IAS-CAAS).

## Author contributions

CL performed the experiment, analyzed data, wrote, and revised the manuscript. RZ investigated, performed the experiment, and collected data. ZZ and CR investigated resources. XW and XH performed estrus detection and collected sample. JM revised the manuscript. XZ and JZ collected samples. RD designed the research and revised the manuscript. MC administrated the project and revised the final manuscript. All authors read and approved the final version.

## Funding

This study was supported by the National Natural Science Foundation of China (31861143012, 31472078, and 31572371), Earmarked Fund for China Agriculture Research System of MOF and MARA (CARS-38), and the Agricultural Science and Technology Innovation Program of China (CAAS-ZDRW202106 and ASTIP-IAS13).

## Conflict of interest

The authors declare that the research was conducted in the absence of any commercial or financial relationships that could be construed as a potential conflict of interest.

## Publisher's note

All claims expressed in this article are solely those of the authors and do not necessarily represent those of their affiliated organizations, or those of the publisher, the editors and the reviewers. Any product that may be evaluated in this article, or claim that may be made by its manufacturer, is not guaranteed or endorsed by the publisher.

## Supplementary material

Supplementary Table S1 Primer sequences employed for qPCR from differentially expressed piRNAs and the target mRNAs in LP vs. FP.

Supplementary Table S2 Length wise distribution data set of piRNAs in FP and LP groups.

Supplementary Table S3 5′−3′ positional nucleotide frequencies (%) data set of piRNAs in FP and LP groups.

Supplementary Table S4 Data set of pi-clusters across chromosomes **(a)** and genomic regions falling within the pi-clusters **(b)**.

Supplementary Table S5 Data set of overlapping pairs and the 5' base frequencies from genome-derived piRNAs in FP **(a)** and LP **(b)**.

Supplementary Table S6 Distribution set of origin of FP and LP-piRNAs among **(a)** Repeats **(b)** Retro repeats.

Supplementary Table S7 Data set of differentially expressed piRNAs in LP vs. FP.

Supplementary Table S8 Biol-information of upregulated piRNAs and the target mRNAs (LP vs. FP).

Supplementary Table S9 Biol-information of downregulated piRNAs and the target mRNAs (LP vs. FP).

Supplementary Table S10 GO terms set of the differentially expressed target mRNAs in LP vs. FP.

Supplementary Table S11 KEGG enrichment of the differentially expressed target mRNAs in LP vs. FP.

Supplementary Table S12 The raw data of qPCR verification for several piRNAs and predicted target genes in LP vs. FP.

Supplementary Figure S1 The relative expression of several piRNAs and predicted target genes in LP vs. FP.
