# Supplementary figures and images for: Expression characteristics of piRNAs in ovine luteal phase and follicular phase ovaries
Source: Front Vet Sci. 2022 Sep 8;9:921868. doi: 10.3389/fvets.2022.921868 (PMC9493120; doi:10.3389/fvets.2022.921868)

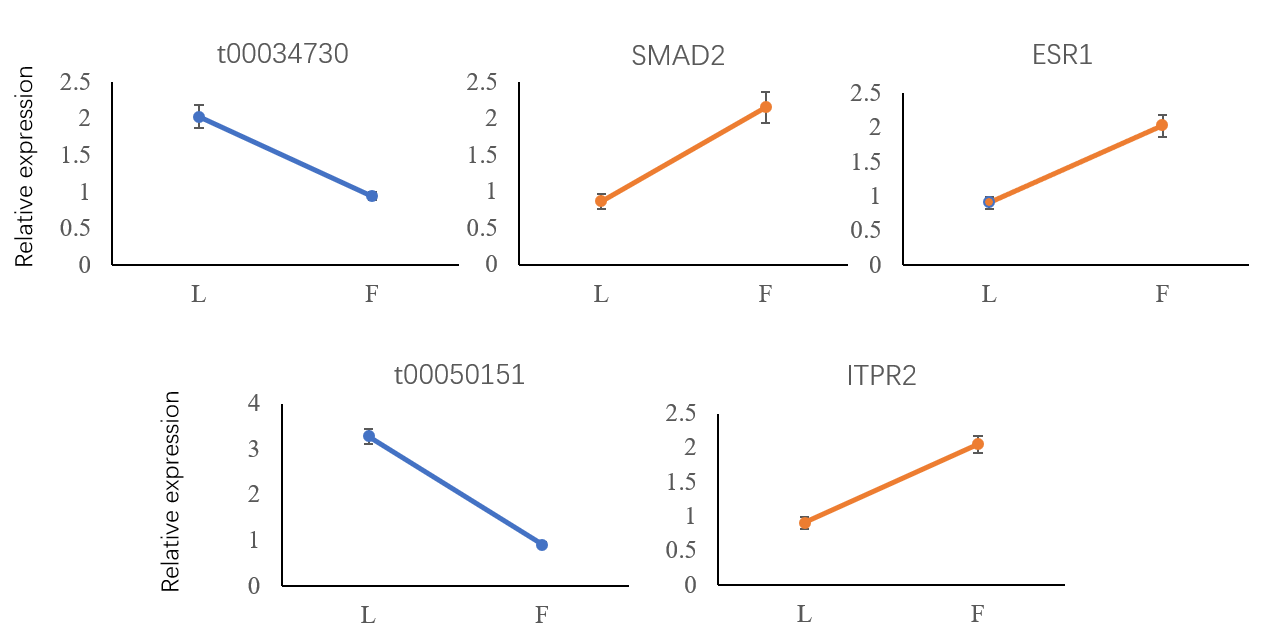

Supplement: Supplementary Figure S1 — The relative expression of several piRNAs and predicted target genes in LP vs. FP. [file Image_1.TIF]
